# Variability and reliability study of overall physical activity and activity intensity levels using 24 h-accelerometry-assessed data

**DOI:** 10.1186/s12889-018-5415-8

**Published:** 2018-04-20

**Authors:** Lina Jaeschke, Astrid Steinbrecher, Stephanie Jeran, Stefan Konigorski, Tobias Pischon

**Affiliations:** 1 Molecular Epidemiology Group, Max Delbrueck Center for Molecular Medicine in the Helmholtz Association (MDC), Robert-Roessle-Strasse 10, 13125 Berlin, Germany; 2 Charité – Universitätsmedizin Berlin, Charitéplatz 1, 10117 Berlin, Germany; 3 DZHK (German Center for Cardiovascular Research), partner site Berlin, Oudenarder Strasse 16, 13347 Berlin, Germany

**Keywords:** Accelerometry, Habitual physical activity, Observational time, Reliability, Variability, Intensities

## Abstract

**Background:**

24 h-accelerometry is now used to objectively assess physical activity (PA) in many observational studies like the German National Cohort; however, PA variability, observational time needed to estimate habitual PA, and reliability are unclear.

**Methods:**

We assessed 24 h-PA of 50 participants using triaxial accelerometers (ActiGraph GT3X+) over 2 weeks. Variability of overall PA and different PA intensities (time in inactivity and in low intensity, moderate, vigorous, and very vigorous PA) between days of assessment or days of the week was quantified using linear mixed-effects and random effects models. We calculated the required number of days to estimate PA, and calculated PA reliability using intraclass correlation coefficients.

**Results:**

Between- and within-person variance accounted for 34.4–45.5% and 54.5–65.6%, respectively, of total variance in overall PA and PA intensities over the 2 weeks. Overall PA and times in low intensity, moderate, and vigorous PA decreased slightly over the first 3 days of assessment. Overall PA (*p* = 0.03), time in inactivity (*p* = 0.003), in low intensity PA (*p* = 0.001), in moderate PA (*p* = 0.02), and in vigorous PA (*p* = 0.04) slightly differed between days of the week, being highest on Wednesday and Friday and lowest on Sunday and Monday, with apparent differences between Saturday and Sunday. In nested random models, the day of the week accounted for < 19% of total variance in the PA parameters. On average, the required number of days to estimate habitual PA was around 1 week, being 7 for overall PA and ranging from 6 to 9 for the PA intensities. Week-to-week reliability was good (intraclass correlation coefficients, range, 0.68–0.82).

**Conclusions:**

Individual PA, as assessed using 24 h-accelerometry, is highly variable between days, but the day of assessment or the day of the week explain only small parts of this variance. Our data indicate that 1 week of assessment is necessary for reliable estimation of habitual PA.

**Electronic supplementary material:**

The online version of this article (10.1186/s12889-018-5415-8) contains supplementary material, which is available to authorized users.

## Background

There is a large body of evidence that regular physical activity (PA) reduces the risk of chronic diseases [1, 2]. The World Health Organization recommends moderate to vigorous PA for at least 150 min per week to prevent noncommunicable diseases [2]. Thus far, PA assessment in observational studies on exposure-disease associations has mostly relied on self-reports, resulting in relatively imprecise, subjective information on intensity and duration of PA [3]. Precise assessment is relevant, since overall PA and time spent in different intensity levels might have different impacts on health.

Today, accelerometers allow objective assessment of overall PA and time spent in different intensity levels under free-living conditions by measuring acceleration of the human body in all three spatial axes [4]. There are a number of epidemiological studies that use accelerometry during waking hours to assess habitual PA, with most of them investigating a 7-day period [5–10]. More recently, 24 h-accelerometry has been introduced in several new large cohort studies, like the German National Cohort [11]. Nevertheless, in most studies focusing on reliability published so far, accelerometers were worn by participants during waking hours only and many have used the older generation of uniaxial accelerometers [9, 10, 12, 13]. Thus, little information is currently available about the variation of overall PA and time spent in different PA intensities on a 24 h day-to-day basis, and about the number of days necessary to estimate habitual PA especially with regard to intensity levels. Such information is important, since in epidemiologic studies one is usually interested in the ‘average’ PA amount, in order to estimate to what extent persons with higher or lower PA levels differ in chronic disease risk. Thus, high within-person variability or low between-person variability will require more repeated assessments to estimate habitual PA.

The aim of this study was to quantify the variability of overall PA and of time spent in different PA intensities assessed through 24 h-accelerometry in a general adult population. We therefore assessed day-to-day variability of overall PA and time spent in different PA intensities over 2 weeks. We further investigated whether these PA parameters systematically differ across the days of assessment or the days of the week. Finally, we assessed the number of days necessary to assess habitual PA and its reliability using 24 h-accelerometry under free-living conditions.

## Methods

### Study population

Data were collected between 2012 and 2014 as part of the ActivE-study in the Molecular Epidemiology Group, Max Delbrueck Center for Molecular Medicine in the Helmholtz Association, Berlin, Germany. The original aim of the ActivE-study was to quantify activity-related energy expenditure based on 24 h-accelerometry assessed PA captured over a 2-week period. For this purpose, 50 participants were recruited as a convenience sample via newspaper, email advertisement, university mailing lists, and public postings, stratified by gender (50:50), age, and body-mass index (BMI) based on a standardized recruitment protocol. Inclusion criteria were age 20–69 years, BMI 18.5–35.0 kg/m^2^, German language skills, and ability to give informed consent. Exclusion criteria were mobility impairments, inability to perform metabolic measurements on the first and last day in the study center, as well as any physiological condition interfering with energy metabolism or weight stability. The study protocol was approved by the ethics committee of the Charité - Universitätsmedizin Berlin and the local data protection officer. All participants gave written informed consent.

### Data collection

Each participant visited the study center twice over a 2-week period. At the first visit, anthropometric measurements were taken and accelerometers were provided to participants. Due to the original aim of the ActivE-study, participants performed a metabolic measurement at the second visit (2 weeks after the first visit). Therefore, they were instructed not to do sports the day before in order not to affect the metabolic measurements. Study center visits were performed on weekdays (Monday to Friday).

PA was assessed using the triaxial accelerometer ActiGraph GT3X+ (ActiGraph LLC, Pensacola, FL, USA), which shows good validity and is now being used in several large cohort studies like the German National Cohort [14–16]. The ActiLife software (version 6.11.0; ActiGraph LLC, Fort Walton Beach, FL, USA) was used to initialize accelerometers, to download activity data, and to determine activity parameters. The raw accelerometer data were sampled with a 100 Hz rate (filter set to default, ‘normal’) using all three spatial axes (and the resulting vector magnitude) and were converted while downloading into 1-s-epochs. The accelerometer was initialized by the study personnel and put on during the first study center visit. Participants were instructed to wear the accelerometer on the right hip for a total assessment time of 2 weeks for all waking and sleeping phases except for water activities, sauna visits, or high contact sports. Since the accelerometers covered 8 days of data collection at a 100 Hz sampling rate, each participant was provided with a second pre-initialized accelerometer, starting automatically at the first day of the second week, thus both accelerometers had 1 day overlap. Participants were instructed to take off the first and put on the second device 1 week after starting the assessment. The second accelerometer was taken off during the second study center visit. Thus, 2 sets of 7-day accelerometer data were obtained per participant. Participants were asked to report any burden due to the 24 h wear of accelerometers.

For the present study, we excluded the first day of assessment, when participants visited the study center and the last day before the metabolic measurement, since activity data on these days may not be representative for a usual day of the week. In sensitivity analyses, we included the last day. We also excluded the day when the first and second accelerometer was exchanged. Therefore, a total of 11 days was available per participant, with six consecutive days for the first and five consecutive days for the second week. Days 1–5 in each week were the same weekdays. Depending on the starting day, participants were assessed on different days of the week.

Participants kept a diary to record sleeping times, accelerometer non-wear time (NWT) periods, and time when participants exchanged the accelerometer over the 2 weeks. We calculated the participants’ NWT based on this information.

For each day, we calculated the ‘vector magnitude counts per minute’ (cpm), averaged over 24 h using the ActiLife software, to quantify overall PA on that day. To determine intensities, activity cpm were converted using the triaxial-derived cut points of the software algorithm ‘Freedson Adult VM3 (2011)’, which classifies 0–2690 cpm as light, 2691–6166 cpm as moderate, 6167–9642 cpm as vigorous, and accelerometer counts ≥9643 cpm as very vigorous PA [16]. Those cut points are equivalent to < 3.0, 3.0–5.99, 6.0–8.99, and ≥ 9.0 metabolic equivalents of task (METs), respectively [16], with, as an example, < 3.0 METs resulting from slow walking and ≥ 9.0 METs being equivalent to vigorous aerobics training [17, 18]. This algorithm used to classify PA intensity levels does not allow the separation of light activity into inactivity and low intensity activity. Therefore, we calculated the 95% percentile of the vector magnitude cpm during all participants’ reported sleeping periods and used this as the cut point to distinguish between inactivity and low intensity activity. Thus, light activity was divided into ‘inactive’ (0–78 cpm) and ‘low’ (79–2690 cpm) intensity activity. We calculated the daily time spent in the five PA intensity levels for each person.

### Statistical analysis

Age, height, weight, and BMI are presented as mean and standard deviation (SD), occupational status group and pre-existing medical conditions as proportions (%), and length and number of NWT periods as median and interquartile range (IQR). Data on overall PA and on time spent in different PA intensities were log-transformed for analyses and are presented as geometric mean (GM) and 95% confidence interval (CI). Differences between sexes were assessed using unpaired t-tests or Mann-Whitney U tests for continuous variables, and using Chi-Square and Fisher’s exact tests for categorical variables.

To calculate the day-to-day variability of overall PA and of time spent in PA intensities over 2 weeks, we estimated the within- (*s*_*w*_^*2*^) and between-person variance (*s*_*b*_^*2*^) using a linear mixed-effects model, with sex as fixed and participant as random effects based on 11 days per participant. Variance components were calculated as percentages of total variance.

To investigate whether total PA and time spent in different PA intensities systematically differ across different days of the week or across the days of assessment, linear mixed-effects models adjusted for sex and including participant as a random effect were applied. Analyses were conducted based on accelerometer data of both weeks with 11 days per participant. Model 1 included day of assessment (1 to 11) as the main fixed effect. Model 2 included day of the week (Monday to Sunday) and week (1 vs. 2) as fixed effects. Model 3 included weekdays (Monday to Friday) versus weekend days (Saturday and Sunday) and week (1 vs. 2) as fixed effects. Calculated least square means and corresponding 95% CI were back-transformed and are presented as GM and 95% CI. *P*-values were calculated for main fixed effects and for the test of trend for day of assessment.

To calculate the number of days necessary to estimate habitual PA, we used the equation provided by Black et al. [19]:

1 $$ D=\frac{r^2}{1-{r}^2}\ast \frac{{s_w}^2}{{s_b}^2} $$

with *D* being the number of consecutive days of PA assessment and *r* being the assumed correlation between the observed and true mean of the PA parameters over the 11 days. The within-to-between-person variance ratio for each PA parameter, *s*_*w*_^*2*^/*s*_*b*_^*2*^, was derived using the baseline model with sex as fixed and participant as random effects with 11 days per participant. We set *r* to be 0.9, indicating that when dividing PA into quintiles, less than 0.1% of all participants would be misclassified into the opposite extreme fraction compared to the true PA (while 75% in the fifth quintile are correctly classified) [20]. By solving Eq. 1 for *r,* we also calculated the correlation between observed and (unknown) true mean of the PA parameters for different numbers of days of assessment.

Finally, we calculated the reliability of PA between week 1 and 2 by calculating intraclass correlation coefficients (ICC) [21, 22], using the underlying between- and within-person variance based on the model with sex as fixed and participant as random effects of the mean daily PA parameter in week 1 and 2 for each participant.

*P*-values presented are 2-tailed and *P* < 0.05 was considered statistically significant. Analyses were performed using SAS® Enterprise Guide®, version 4.3 (SAS Institute Inc., Cary, NC).

## Results

Overall PA, as averaged over the 2-week period over all participants, was 437.0 cpm (Table 1). Median time spent in inactivity, and low intensity, moderate, vigorous, and very vigorous activity was 1186.8 min/day, 127.1 min/day, 95.7 min/day, 14.4 min/day, and 3.8 min/day, respectively. There were only slight, non-significant differences between men and women in the PA parameters.

**Table 1:** Characteristics of the Study Population, ActivE-Study, 2012–2014

	Men (*n* = 25)	Women (*n* = 25)	Total (*n* = 50)	Test for sex^a^
Characteristics of study population				*p*
Age, years, mean (SD)	49.9 (13.7)	40.0 (14.6)	45.0 (14.9)	0.02
Height, cm, mean (SD)	181.0 (6.0)	167.5 (6.5)	174.3 (9.2)	<.0001
Body weight, kg, mean (SD)	87.8 (12.1)	72.5 (12.7)	80.2 (14.5)	<.0001
BMI, kg/m^2^, mean (SD)	26.8 (3.5)	25.9 (4.6)	26.4 (4.1)	0.42
Occupation, %				0.04
Full time	56	56	56	
Part time	8	32	20	
Unemployed	36	12	24	
Diabetes mellitus, %	0	0	0	–
Hypertension, %	16	4	10	0.34
Coronary artery disease, %	4	0	2	1.00
Cancer, %	4	0	2	1.00
Overall PA, cpm, GM (95% CI)^b^	426.8 (382.8, 475.8)	447.4 (401.3, 498.8)	437.0 (404.8, 471.6)	0.54
Time in inactivity, min/d, GM (95% CI)^b^	1196.3 (1172.3, 1220.7)	1177.5 (1153.9, 1201.5)	1186.8 (1169.9, 1204.0)	0.27
Time in low intensity activity, min/d, GM (95% CI)^b^	118.6 (107.0, 131.3)	136.4 (123.1, 151.0)	127.1 (118.1, 136.9)	0.06
Time in moderate activity, min/d, GM (95% CI)^b^	93.3 (84.5, 102.9)	98.2 (89.0, 108.4)	95.7 (89.3, 102.6)	0.46
Time in vigorous activity, min/d, GM (95% CI)^b^	14.7 (11.8, 18.4)	14.1 (11.3, 17.5)	14.4 (12.3, 16.8)	0.76
Time in very vigorous activity, min/d, GM (95% CI)^b^	4.0 (2.9, 5.6)	3.6 (2.6, 5.0)	3.8 (3.1, 4.8)	0.62
Average NWT per participant (over 11 days), min, median (IQR)^c^	254.5 (124.0, 392.0)	205.0 (115.0, 270,0)	215.0 (120.0, 338.0)	0.23
Number of NWT periods per participant (in 11 days), median (IQR)^c^	8.0 (6.5, 11.5)	10.0 (7.0, 12.0)	9.0 (7.0, 12.0)	0.53
NWT per day, min, median (IQR)^c^	15.0 (0.0, 30.0)	13.0 (4.0, 23.0)	13.0 (0.0, 25.0)	0.90
Number of NWT periods per day, median (IQR)^c^	1.0 (0.0, 1.0)	1.0 (1.0, 1.0)	1.0 (0.0, 1.0)	0.22

*BMI* body-mass index, *cpm* counts per minute, *GM* geometric mean, *IQR* interquartile range, *NWT* non-wear time, *PA* physical activity, *SD* standard deviation, *95% CI* 95% confidence interval

^a^continuous variables, normally distributed: t-test; continuous variables, not normally distributed: Mann-Whitney U test; categorical variables: Chi-Square test/Fisher’s exact test

^b^analyses were performed using log-transformed physical activity data

^c^derived from participants’ activity diaries

None of the participants reported complaints during waking or sleeping times that prevented continuous wearing of the accelerometers. According to their diaries, per day, participants took off the accelerometer on average 1.0 (IQR; 0.0, 1.0) time for a medium length of 13.0 (0.0, 25.0) minutes (Table 1). There were no significant differences in NWT between men and women. NWT periods reported in the diaries were similar to the accelerometer data in terms of daytime and length of NWT periods (data not shown). Due to the small numbers and short lengths of NWT periods, we included the NWT in all analyses. Since we did not observe differences in PA or NWT between men and women, we combined both sexes in our analyses.

For overall PA and for the time spent in different PA intensities, between-person variance accounted for 34.4–45.5% of total variance, whereas within-person (day-to-day) variance accounted for 54.5–65.6% (Table 2).

**Table 2:** PA within- and between-person variance and number of days to assess habitual PA, total (*N* = 50)

			Percent of total variance		
PA parameter^a^	Within-person variance	Between-person variance	Within-person variance	Between-person variance	Number of days
	s_w_^2^	s_b_^2^	s_w_^2^	s_b_^2^	D^b^
Overall PA, cpm	0.09593	0.06317	60.3	39.7	7
Time in inactivity, min/d	0.00273	0.00225	54.9	45.1	6
Time in low intensity activity, min/d	0.06727	0.05627	54.5	45.5	6
Time in moderate activity, min/d	0.09604	0.05033	65.6	34.4	9
Time in vigorous activity, min/d	0.28890	0.21890	56.9	43.1	6
Time in very vigorous activity, min/d	0.57190	0.34590	62.3	37.7	8

*cpm* counts per minute, *D* number of days to assess physical activity based on a given r (assumed correlation between observed and true mean of physical activity parameter) [19], *PA* physical activity, *s*_*b*_^*2*^ between-person variance over 11 days of physical activity, *s*_*w*_^*2*^ within-person variance over 11 days of physical activity

^a^all analyses were performed using log-transformed data

^b^D is calculated based on an assumed correlation between observed and true mean physical activity of *r* = 0.9 and is rounded up to the nearest full number of days

**Fig. 1:**
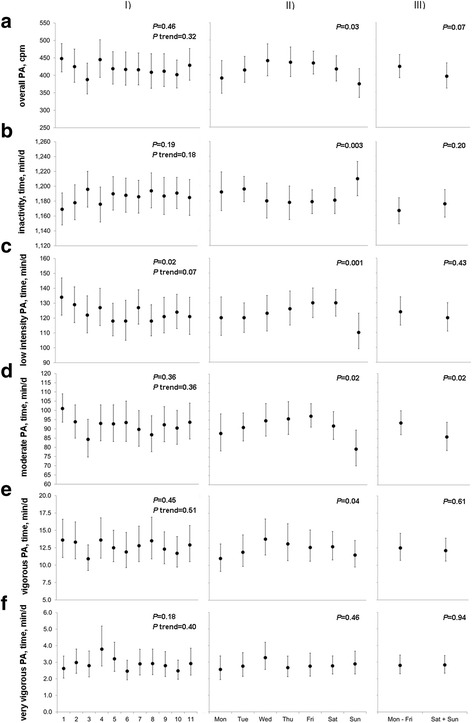
Physical activity according to day of assessment, day of the week, or weekend versus weekday. Results of linear mixed-effects models with adjustment for sex (Panel **I**) or week and sex (Panels **II** and **III**). Dots indicate geometric least square means and error bars 95% confidence intervals for overall physical activity (PA) (counts per minute, cpm, Panel **a**), time in minutes in inactivity (Panel **b**), and in low intensity (Panel **c**), moderate (Panel **d**), vigorous (Panel **e**), or very vigorous activity per day (Panel **f**). *P*-values presented refer to the main fixed effects day of assessment (Panel **I**; overall *p*-value and p-trend), day of the week (Panel **II**), or weekday versus weekend day (Panel **III**)

We next investigated whether overall PA and time spent in different PA intensities systematically differ across the days of assessment, days of the week, or between weekdays and weekend days (Fig. 1). Overall, there were only relatively small differences in all PA parameters in these analyses. For day of assessment, overall PA, and time in low intensity, moderate, and vigorous activity were highest at the first day and (except for time in low intensity activity) lowest on the third day. The pattern for time in inactivity was complementary to these observations. Over the days of the week, overall PA, and time in low intensity, moderate, and vigorous PA differed, being highest on Wednesday and Friday and lowest on Monday and Sunday, with apparent differences between Saturday and Sunday (Fig. 1). Thus, overall PA was lower and time in low intensity, moderate, and vigorous PA were shorter on Sunday than on Saturday. The opposite pattern was found for time in inactivity. We observed only slight differences in time in very vigorous activity over the days of the week. Time in moderate activity was significantly lower on weekend days compared to weekdays (Fig. 1). Overall PA tended to be lower on weekend days than on weekdays, while time in inactivity was slightly longer on weekend days when compared to weekdays. However, these differences were not statistically significant.

In a model with participant and day of the week as consecutively nested random instead of fixed effects, the day of the week accounted for 2.1% of total variance observed in overall PA, 3.8% in time in inactivity, 9.6% in time in low intensity activity, 9.4% in time in moderate activity, 0.3% in time in vigorous activity, and 0.0% for time in very vigorous activity. Further, assuming participant, week (1 vs. 2), and the distinction between weekdays (Monday to Friday) and weekend days (Saturday and Sunday) as consecutively nested random instead of fixed effects, the week-vs-weekend-day-effect explained 13.9%, 11.9%, 17.5%, 18.8%, 5.7%, and 7.2% of total variance, respectively.

The number of days, *D*, necessary to validly characterize habitual PA was lowest for time in inactivity, and for time in low intensity and vigorous activity (*D* = 6 days), and highest for time in moderate activity (*D* = 9) (Table 2). The correlation between observed and true PA, *r*, estimated depending on the number of days of PA assessments, is summarized in an additional file (see Additional file 1). These data indicate that habitual PA is reasonably estimated with repeated measurements over a period of around 1 week.

Finally, we assessed the week-to-week reliability of habitual PA when estimated based on measurements over an approximately 1-week period. The ICCs for the different PA parameters were in the range of 0.68 (for time in moderate activity) to 0.82 (for time in vigorous activity) indicating good to excellent reliability [22]. The ICC for overall PA was 0.75 (Table 3)

**Table 3:** Within- and between-person variance and week-to-week reliability of habitual physical activity, total (*N* = 48)

			Percent of total variance			
PA parameter^a^	week 1	week 2	within-person variance	between-person variance	ICC between week 1 and 2	
	GM	GM	s_w_^2^	s_b_^2^	ICC	95% CI
Overall PA, cpm	438.0	421.3	25.3	74.7	0.75	0.60, 0.85
Time in inactivity, min/d	1186.0	1192.1	25.3	74.7	0.76	0.61, 0.86
Time in low intensity activity, min/d	127.2	124.3	25.0	75.0	0.78	0.64, 0.87
Time in moderate activity, min/d	96.2	91.9	33.3	66.7	0.68	0.49, 0.80
Time in vigorous activity, min/d	14.0	13.8	18.2	81.8	0.82	0.70, 0.89
Time in very vigorous activity, min/d	3.7	3.4	32.0	68.0	0.69	0.51, 0.81

*cpm* counts per minute, *ICC* intraclass correlation coefficient, *GM* geometric mean, *PA* physical activity, *s*_*b*_^*2*^ between-person variance of physical activity between week 1 and 2, *s*_*w*_^*2*^ within-person variance of physical activity between week 1 and 2, *95% CI* 95% confidence interval

^a^all analyses were performed using log-transformed data

## Discussion

In this study, we found high within-person (day-to-day) variability in overall PA and in times spent in inactivity, and in low intensity, moderate, vigorous, or very vigorous activity as assessed using 24 h-accelerometry over 2 weeks in the general adult population. We found significant differences across the days of assessment and the days of the week across all study participants. However, overall, these systematic differences were relatively small and the day of assessment or the day of the week explained only little of the total variance in the PA parameters. Our data indicate that for suitable characterization of habitual PA, around 1 week of assessment is necessary. Comparing PA between two consecutive weeks (using a 5–6 day average in each week), habitual PA showed good week-to-week reliability.

Our results indicate that the day-to-day within-person variance of 24 h PA over 2 weeks is larger than between-person variance, accounting for around 60% of total variance in overall PA and in time spent in different PA intensities. A study by Matthews et al. found the between-variance to account for the largest variance component for overall PA (around 60%) and the within-person variance to account for the largest proportion (60%) of total variance in time spent in inactivity [12]. However, direct comparability with the finding in our study is limited because in the study by Matthews et al. PA was assessed during waking hours only, whereas, in our study, it was assessed over 24 h.

We expected daily overall PA to decrease with time under observation because participants would be more active than normal on the first days, since they are aware of being studied. However, we did not observe such a trend in the PA parameters over the days of assessment. Overall, differences in the day of assessment explained only little of the total variance in PA.

Average overall PA, time in inactivity, and in low intensity, moderate, and vigorous activity tended to differ slightly between the days of the week. This might be due to the fact that household activities (being mostly of low intensity) as well as sports and exercise (moderate and vigorous activity), which are more planned behaviors and thus better predictable and less variable throughout the week, mainly fall in these PA intensities [17, 23]. However, altogether, variability in mean PA over the days of the week was relatively small, which is confirmed by the finding that the day of the week explained less than 10% of the total variance in all PA parameters. These observations are consistent with results by Matthews et al. and Tudor-Locke et al., who found the day of the week to explain less than 8% [12, 24]. Further, these authors also found PA to be lowest on Sunday, which is in agreement with our study [12, 24, 25].

There are recommendations to require at least 1 weekend day when assessing PA using accelerometers, since PA may differ between weekdays and weekend days [4, 25–27]. In our study, we observed only small differences in PA between weekdays and weekend days. However, we also found that Saturday was different from Sunday, as reported in other studies [12, 24]. While Saturday was more comparable with weekdays in terms of PA, activity levels were lower and the inactivity level was higher on Sundays as compared to the other days of the week. Thus, our data indicate that inclusion of both, Saturday and Sunday is required to obtain unbiased PA estimates, since inclusion of either, Saturday or Sunday may result in over- or underestimation of weekend PA.

We used a formula by Black et al. to calculate the number of days needed for reliable assessment of PA. This formula was originally developed to calculate the number of 24 h dietary recalls needed to estimate energy intake in infants [19, 20]. Today, this formula has been used in several other studies and is well-established in the field of nutritional epidemiology across all ages [28–31], although to our knowledge it hasn’t been used in the field of PA. The Black formula is based on the within-to-between-person-variance ratio and on the assumed correlation between observed and true levels of PA. As such, the formula should be generalizable to the field of PA. Previous studies have used the Spearman-Brown prophecy formula, which determines the number of days needed to obtain a desired reliability. As such, it relies on the ICC [32]. However, the applicability of the Spearman-Brown formula in the field of PA has been criticized because it depends on the assumption of compound symmetry, and this may not hold true for PA data [32]. Nevertheless, when we applied the Spearman-Brown prophecy formula in our sensitivity analyses, we obtained similar results to the Black formula (data not shown). We therefore speculate whether potential violations of the underlying assumptions of these formulas may not substantively affect the results, although future studies are warranted to investigate this in detail.

A recent study by Wolff-Hughes et al. investigated the number of accelerometer monitoring days needed for stable group-level estimates of activity [10]. They concluded that a single day of assessment may be sufficient to measure mean group total PA and of time in activity intensities [10]. However, it should be noted that the study by Wolff-Hughes et al. focused on the question on how many days are needed to estimate the mean PA in a group; thus, their approach did not take the number of days necessary to obtain reliable mean within-person PA into account. While a single day of assessment may well estimate the true mean group PA, it is unlikely that a single day provides a valid estimate of true between-person variance. In fact, our study suggests that approximately 1 week of assessment is necessary to reliably classify persons based on their ‘average’ PA. Beyond these differences, Wolff-Hughes et al. used an older generation of uniaxial accelerometers over 1 week during waking hours only, whereas we used triaxial devices over 24 h/day and 2 weeks. Thus, comparability between both studies and their conclusions is limited.

When estimated based on approximately 1 week of daily assessment (5–6 days), we found high week-to-week reliability for habitual PA. These findings are similar to reports by Sirard et al., assessing 2 weeks 1 to 4 weeks apart, and to results seen in older adults [9, 13]. These findings have implications for researchers designing new studies with accelerometry-based PA assessment: Depending on the PA parameter of interest, the number of days of 24 h-accelerometry assessment for a reliable habitual PA estimation can be considered carefully. It is important to note that this number refers to days with complete PA assessment. Thus, any extra days to put on and take off of the accelerometer should also be taken into account. Alternatively, for those studies, where PA data have already been collected, our data provide information on the reliability of PA for a given number of days of assessment. Since low reliability usually attenuates exposure-disease associations towards the null, the ICCs provided in our study may also allow to calculate deattenuated estimates of observed relative risks [33]. These are important implications for obtaining reliable relative risk estimates in cohort studies.

A strength of our study was the focus on participants under free-living conditions. The short NWT (and high feasibility of 24 h wear of accelerometers) observed in our study population resulted in almost unaffected PA parameters and allowed a meaningful estimation of variability and reliability of habitual daily PA. In contrast to most other studies, especially those regarding variability of PA, we had data on PA recorded over 24 h per day for 2 weeks, thus covering the entire PA spectrum [12, 24, 25]. Nevertheless, our study has some limitations. The sample size was relatively small, and our study population is probably not fully representative of the general adult population. Observed time in moderate activity might appear to be slightly longer than observed in other studies that used accelerometry during waking hours [6, 12], although direct comparability with studies that assessed PA during waking phases only is limited, since we assessed 24 h-PA. Further, the main focus of our study was to assess variability and reliability of PA, and we do not expect these to be substantially affected by a slightly higher average group PA level. Nevertheless, further studies are warranted to examine variability and reliability of PA in other populations, such as persons with different phenotypes (e.g., extreme obesity; narrower age ranges or younger or older ages; diseased populations). We determined the accelerometer NWT based on the participants’ diary, since currently available accelerometer-based NWT algorithms were developed for data captured during waking hours only and may thus not be suitable for NWT in 24 h-accelerometry [34]. We included the observed NWT periods in our analyses, since these were seldom and short, acknowledging that this may only slightly overestimate time in inactivity. Our findings of no substantial association between the day of the week and average PA across the entire population does not rule out systematic within-person differences in PA between weekdays. However, if such effects are present, our results suggest that they are randomly distributed across the population. Participants were asked to pursue their normal daily routine over the 2 weeks and there was no evidence for an observation bias in our study; nevertheless, study participation in general and the use of activity diaries might have influenced usual behavior of the participants. Since the algorithm used to determine PA intensities did not allow distinguishing between inactivity and light activity, we derived a new cut point to enable this separation. We did not differentiate sedentary behavior from light activity intensity in our study. Studies have shown that sedentary behavior may be an additional risk factor for poor health outcomes beyond physical inactivity [35–41]. Therefore, future studies are warranted to assess the reliability of sedentary behavior assessment based on 24 h accelerometry. It also should be noted that we assessed PA over 2 consecutive weeks and observed high reliability, which is consistent with published data; however we cannot rule out that reliability may be lower over longer time periods, different seasons, or when weeks are randomly selected (instead of consecutively) [32, 42–45]. Finally, our assessment did not exactly encompass 2 full weeks, since we had to delete days from each week for technical reasons, as described. As we had to delete day 12 of assessment, comparison between week 1 and 2 was unbalanced. The ‘week-to-week’ reliability was therefore derived from six versus five days included in weeks 1 and 2, respectively. However, since we compared the mean daily activity of each week, i.e., PA averaged over the days in each week per participant, we assume the reliability of 24 h-accelerometry based PA not to have been substantially biased by not comparing two full weeks. Further, as sensitivity analyses, we performed all analyses including day 12 allowing for comparison of two more complete weeks using a balanced design and results were similar (data not shown).

## Conclusion

In conclusion, our study showed a high within-person day-to-day variability in objective PA based on 24 h-accelerometry, but neither the day of assessment nor the day of the week substantially explained this observed variance. Assessing PA over a 1-week period (including a 5–6 day mean) allows a reliable estimation of average individual overall PA and of mean times in different PA intensities, and is thus a suitable approach in epidemiological studies. In this context, PA duration and intensity may have distinct effects on the disease risk, highlighting the epidemiological relevance of accelerometry that allows for the reliable assessment of both, PA duration and intensity.

## Additional file

Additional file 1: Correlations between observed and true physical activity based on a given number of days of assessment. Summarizes the correlation, *r*, between observed and (unknown) true mean of six physical activity (PA) parameters, estimated depending on the number of days, *D*, of repeated PA assessments. Calculation is based on an equation by Black et al. using between-person, s_b_^2^, and within-person variance, s_w_^2^, solved for r [19]. (DOCX 20 kb)

## Abbreviations

BMI: Body-mass index
CI: Confidence interval
cpm: Counts per minute
GM: Geometric mean
ICC: Intraclass correlation coefficients
IQR: Interquartile range
METs: Metabolic equivalents of task
NWT: Non-wear time
PA: Physical activity
s_b_^2^: Between-person variance
SD: Standard deviation
s_w_^2^: Within-person variance
