# Supplementary material for: Variability and reliability study of overall physical activity and activity intensity levels using 24 h-accelerometry-assessed data
Source: BMC Public Health. 2018 Apr 20;18:530. doi: 10.1186/s12889-018-5415-8 (PMC5910625; doi:10.1186/s12889-018-5415-8)
Supplement: Supplementary file 1 — Correlations between observed and true physical activity based on a given number of days of assessment. Summarizes the correlation, r, between observed and (unknown) true mean of six physical activity (PA) parameters, estimated depending on the number of days, D, of repeated PA assessments. Calculation is based on an equation by Black et al. using between-person, sb2, and within-person variance, sw2, solved for r [19]. (DOCX 20 kb) [file 12889_2018_5415_MOESM1_ESM.docx]

Additional file 1: Correlations between observed and true physical activity based on a given number of days of assessment

| **PA parameter^a^** | **Within-person variance** | **Between-person variance** |  | **r** | | | | | | | | | | |
| --- | --- | --- | --- | --- | --- | --- | --- | --- | --- | --- | --- | --- | --- | --- |
|  | **s_w_²** | **s_b_²** | **s_w_²/s_b_²** | **d=1** | **d=2** | **d=3** | **d=4** | **d=5** | **d=6** | **d=7** | **d=8** | **d=9** | **d=10** | **d=11** |
|  |  |  |  |  |  |  |  |  |  |  |  |  |  |  |
| overall PA, cpm | 0.09593 | 0.06317 | 1.52 | 0.63 | 0.75 | 0.81 | 0.85 | 0.88 | 0.89 | 0.91 | 0.92 | 0.93 | 0.93 | 0.94 |
| time in inactivity, min/d | 0.00273 | 0.00225 | 1.22 | 0.67 | 0.79 | 0.84 | 0.88 | 0.90 | 0.91 | 0.92 | 0.93 | 0.94 | 0.94 | 0.95 |
| time in low intensity activity, min/d | 0.06727 | 0.05627 | 1.20 | 0.67 | 0.79 | 0.85 | 0.88 | 0.90 | 0.91 | 0.92 | 0.93 | 0.94 | 0.95 | 0.95 |
| time in moderate activity, min/d | 0.09604 | 0.05033 | 1.91 | 0.59 | 0.72 | 0.78 | 0.82 | 0.85 | 0.87 | 0.89 | 0.90 | 0.91 | 0.92 | 0.92 |
| time in vigorous activity, min/d | 0.28890 | 0.21890 | 1.32 | 0.66 | 0.78 | 0.83 | 0.87 | 0.89 | 0.91 | 0.92 | 0.93 | 0.93 | 0.94 | 0.94 |
| time in very vigorous activity, min/d | 0.57190 | 0.34590 | 1.65 | 0.61 | 0.74 | 0.80 | 0.84 | 0.87 | 0.89 | 0.90 | 0.91 | 0.92 | 0.93 | 0.93 |
|  |  |  |  |  |  |  |  |  |  |  |  |  |  |  |

cpm, counts per minute; d, number of days of physical activity assessment; PA, physical activity; r, correlation between observed and true mean of physical activity parameter based on a given d, adapted from [19]; s_b_², between-person variance over 11 days of physical activity; s_w_², within-person variance over 11 days of physical activity
^a^all analyses were performed using log-transformed data
